# UV Index Does Not Predict Ocular Ultraviolet Exposure

**DOI:** 10.1167/tvst.10.7.1

**Published:** 2021-06-01

**Authors:** Natsuko Hatsusaka, Yusuke Seki, Norihiro Mita, Yuki Ukai, Hisanori Miyashita, Eri Kubo, David Sliney, Hiroshi Sasaki

**Affiliations:** 1 Department of Ophthalmology, School of Medicine, Kanazawa Medical University, Kahoku, Ishikawa, Japan; 2 Division of Vision Research for Environmental Health, Project Research Center, Medical Research Institute, Kanazawa Medical University, Kahoku, Ishikawa, Japan; 3 Department of Environmental Health Science and Engineering, Johns Hopkins University Bloomberg School of Public Health, Baltimore, MD, USA

**Keywords:** ocular UV exposure, UV index, ocular UV index, solar altitude, UVB irradiance

## Abstract

**Purpose:**

The ultraviolet index (UVI), available online, is an international linear scale of levels from 0 to 13+ that warns about the risk of sunburn; however, it does not address the risk to eyes. Our purpose was to develop a useful instrument to warn the public against ocular ultraviolet (OUV) exposure and to serve as a tool for researching UV-induced ocular diseases.

**Methods:**

A rotating model head that included ultraviolet B (UVB) sensors documented UV irradiance at the crown and at the eyes spanning eight azimuths from sunrise to sunset under different climatic conditions in each season. The dose intensities obtained were compared with their respective UVI levels. Doses to the eyes were mathematically transformed to develop an OUV index with linear levels from 0 to 13+, similar to the UVI. Then, readings from both instruments were compared.

**Results:**

UV exposure at the crown increases with solar culmination, whereas that to the eye is greater under low rather than maximum solar altitude. The OUV index levels were higher than recorded UVI levels in the summer under low solar altitude in the early morning and mid- to late afternoon and were markedly higher all day in winter when solar altitude remains low.

**Conclusions:**

The UVI does not provide sufficient warning about the risks of ocular UV damage. The proposed OUV index is a useful instrument to warn the public against OUV exposure and to serve as a tool for researching UV-induced ocular diseases.

**Translational Relevance:**

The OUV index is useful to prevent ocular UV-related diseases.

## Introduction

It is known that ultraviolet (UV) radiation exposure can cause acute ocular diseases such as UV keratitis (photokeratitis, snow blindness)^1^^,^^2^ and chronic ocular diseases, including cataracts,^3^^–^^6^ pterygium,^7^^–^^9^ pinguecula,^10^^–^^12^ and climatic droplet keratopathy.^11^^,^^13^^,^^14^ Eyes may be safeguarded from UV exposure by sunglasses, particularly UV blocking spectacles or contact lenses, and by hats or shades such as parasols, yet few people are proactive in safeguarding their eyes, possibly due to low awareness of the risks.^15^^,^^16^ However, awareness of the effects of UV light on skin, including sunburn,^17^^–^^19^ wrinkles and discoloration,^17^^,^^19^^,^^20^ and cancer,^17^^,^^21^^–^^23^ is high, and people often take precautions to protect their skin.

The ultraviolet index (UVI) is an international standard scale of sunburn-inducing UV at a particular place and time.^24^^,^^25^ The UVI is a linear scale ranging from 0 to 13+ that correlates with the severity of risk of UV-induced damage to light-skinned human skin relative to irradiance and wavelength. In practice, the health risks are further complicated by other factors.^26^^,^^27^ It is necessary to consider not only direct rays from the sun but also light scattered by clouds and reflected from surfaces.^28^ It is possible for anybody to obtain UVI information in real time online. The UVI is recognized as an efficient educational tool for communicating UV radiation levels to people in their daily lives. Terrestrial UV irradiance differs with solar altitude. Sasaki et al.^29^^,^^30^ reported that ocular exposure is maximum when the sun is directly to the front at approximately 40° of solar altitude. Their study showed that ocular UV irradiance was not necessarily proportionate to skin UV irradiance; therefore, the UVI did not appear to be a good indicator for the risk of UV-induced ocular disease. Hu et al.^31^ also measured solar UV exposure to eyes using a mannequin UV measuring instrument. They reported results similar to those of Sasaki et al.,^29^^,^^30^ despite there being some differences in the measurements of UV wavelength bands and the mannequin face shape (e.g., eye depth) between the studies.

In the present study, a mannequin type of dosimeter system with ultraviolet B (UVB) sensors was employed to investigate the relationships between UVI levels and UV irradiance at the eye and at the crown of the head in order to create an ocular ultraviolet index (OUVI) and determine how it relates to the UVI.

## Methods

### Use of Mannequins to Measure UV Exposure

**Figure 1.**
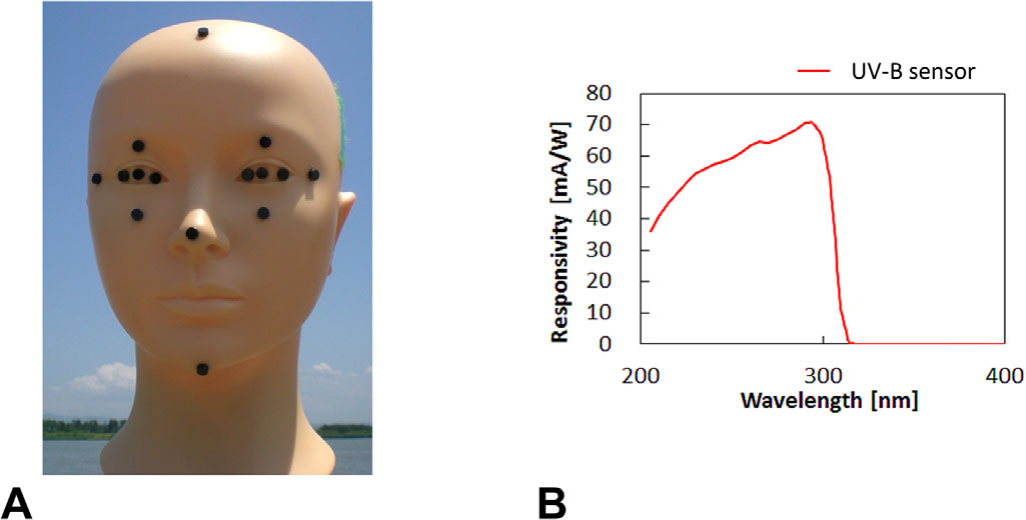
Mannequin UV exposure system with 16 UVB sensors on a dummy head simulating a woman 170 cm tall. (A) Original, full-UV spectral response in an earlier study compared to (B) the spectral sensitivity characteristics of the UVB sensor (absolute value) in this study.

**Figure 2.**
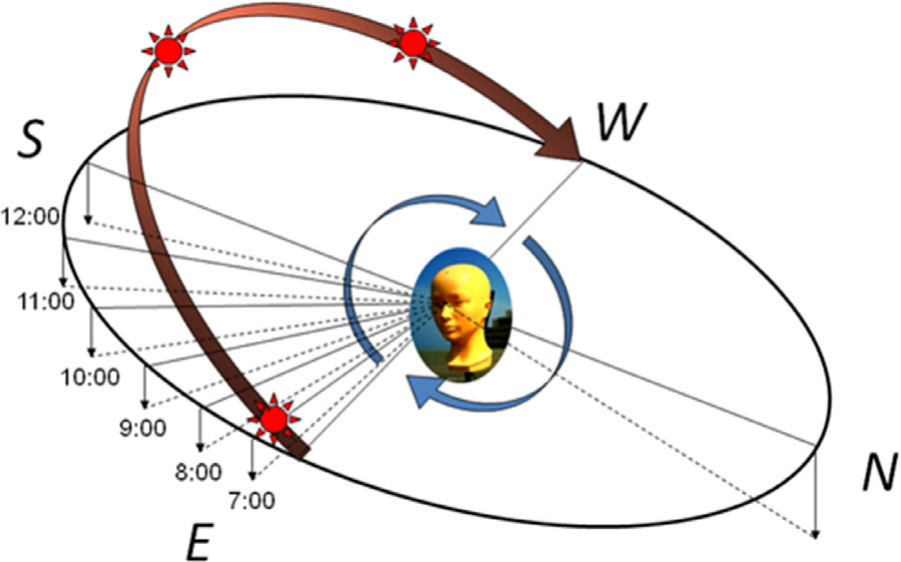
Measurement method. The head rotated and measured UV for 20 seconds at eight azimuths every hour.

The mannequin type of dosimeter system included 16 ultraviolet B (UVB) sensors (MUS-01 InGaN sensor, 4 × 4 × 1.5 mm, sensitivity 280–310 nm; ALGAN K.K., Kyoto, Japan) and a dummy bald head with an adult Japanese female face (Figs. 1A, 1B). Sensors were set in corresponding locations on either side of the face; three were placed on the transverse *x*-axis inside each eye socket (nasal, pupil, temporal) and one on each temple on the same axis; four were set on the sagittal *y*-axis of the head (crown, forehead, tip of the nose, chin); and two were placed on the sagittal *y*-axis of each eye (brow, cheek). The mannequin system represented a woman 170 cm tall with her head tilted down and a sight line 15° below the horizontal to simulate a natural posture.^29^^,^^32^^,^^33^ The mannequin was set on an equatorial telescope mount so it could be rotated. UV exposure was measured at eight azimuths (north, northeast, east, southeast, south, southwest, west, northwest) on clear days and on a day with cloudy skies. The system rotated to a position where the dummy eyes were facing the sun, as well as through the eight azimuths, stopping at each position for 20 seconds to record values before moving to the next every hour from sunrise to sunset (Fig. 2).

### Measurement Locations and Dates

**Figure 3.**
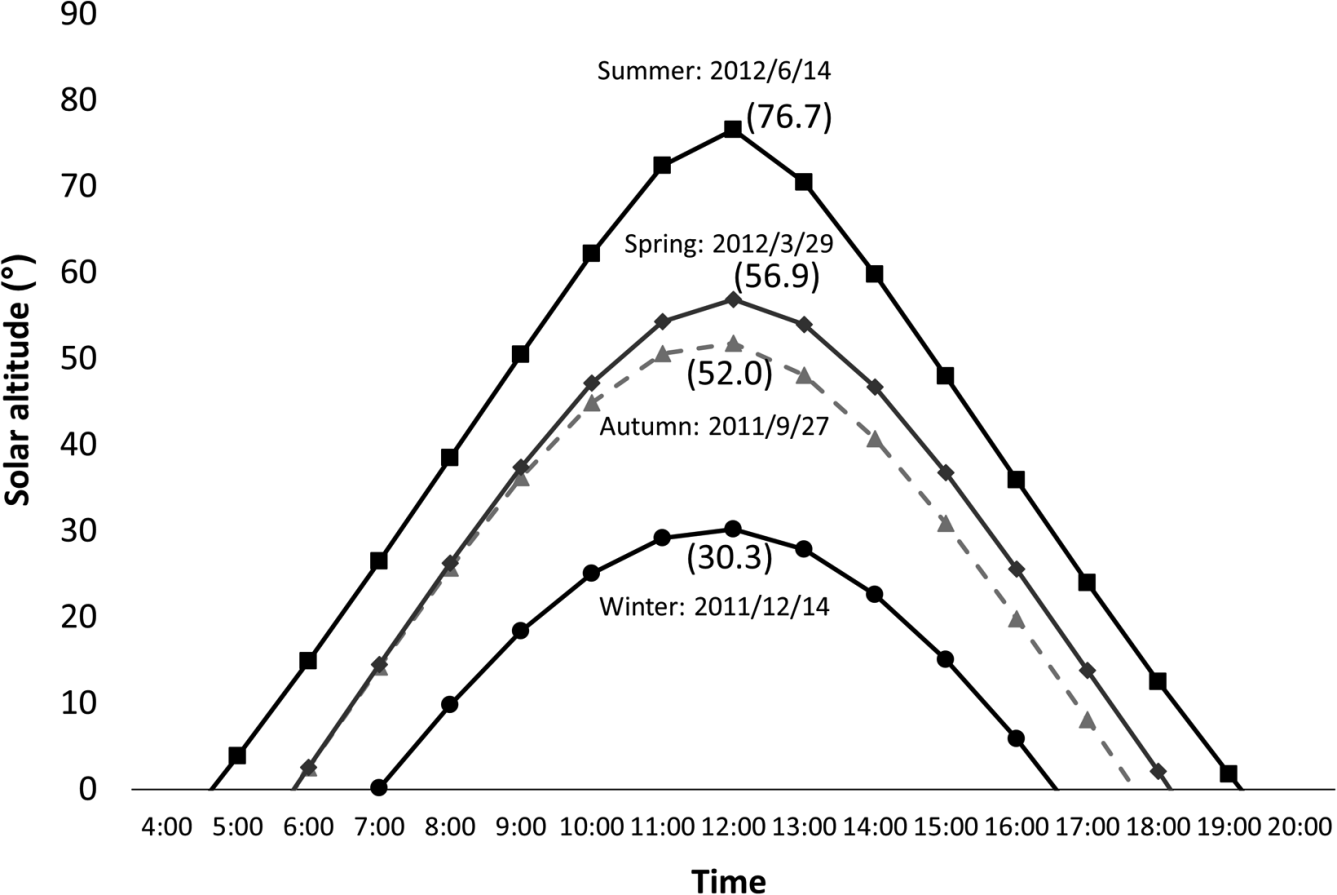
Solar altitudes (*y*-axis) in Kanazawa, Japan (latitude 36.66°N, longitude 136.65°E) by time (*x*-axis) on September 27, 2011, and December 14, 2011, and on March 29, 2012, and June 14, 2012. Values in parentheses show solar culmination altitudes.

Measurements were made on a gray, urethane-coated, concrete roof (∼10% UV reflectance) of Kanazawa Medical University (latitude 36.66°N, longitude 136.65°E, elevation 50 m) on September 27, 2011, and December 14, 2011, and on March 29, 2012, and June 14, 2012, with solar culmination altitudes of 52.0°, 30.3°, 56.9°, and 76.7°, respectively, based on National Astronomical Observatory of Japan data (Fig. 3). Weather, maximum temperature, sunlight hours, culmination altitude, and maximum UVI data were obtained at the time of UV measurements from the Japan Meteorological Agency and National Astronomical Observatory of Japan.^34^^,^^35^ The weather was fine and there was a clear sky on each day; the maximum UVI levels on the measurement days were 5, 1, 5, and 7, respectively (Table 1) (Appendices A to D).

**Table 1.** Survey Conditions

Season	Date	Weather	Maximum Temperature (°C)	Sunlight (h)	Culmination Altitude (°)	Maximum UVI
Spring	March 29, 2012	Clear sky	14.7	11.0	56.9	5
Summer	June 14, 2012	Clear sky	26.1	13.4	76.7	7
Autumn	September 27, 2011	Clear sky	26.3	11.2	52.0	5
Winter	December 14, 2011	Clear sky	14.3	6.4	30.3	1

### Creation of the OUVI

**Figure 4.**
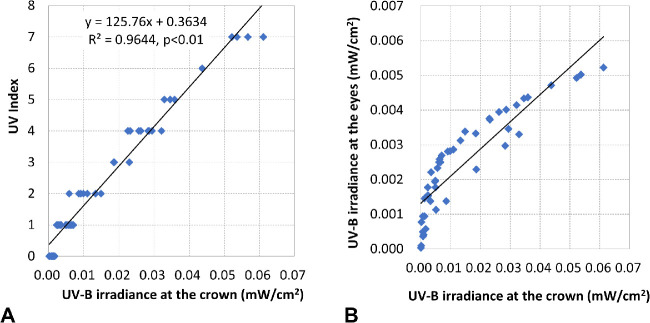
UV index scale and UVB exposure. (A) Correlation between UV index and UV irradiance at the crown of the head. UV exposure correlated highly with UV index. B: Correlation between UVB irradiance at the crown of the head and the eyes.

The UVB values obtained by the sensor on the crown of the head, which remained pointing to the sky (90°) at all times, were highly correlated with UVI levels (Fig. 4A), indicating that it was possible to determine UVI levels from crown exposure in real time. Accordingly, it would be possible to determine an OUVI using the ocular UVB exposure, which was approximately 1/10 of that at the crown of the head (Fig. 4B). We used the value of 10-fold ocular UVB exposure and defined the OUVI based on a linear scale from 0 to 13+, similar to that of the UVI. The OUVI was calculated using the following approximate linear equation derived from Figure 4:

$$\begin{array}{ccc} \mathrm{OUVI} & = & 125.76\times \left(\mathrm{ocular}\;\mathrm{UVB}\;\mathrm{irradiance}\times 10\right)\\ \; & + & 0.3634 \end{array}$$

The OUVI was calculated for each hour and compared with UVI levels.

### Analysis

SPSS Statistics (IBM Corporation, Armonk, NY) was used for statistical analysis, and Pearson's correlation coefficients were determined to summarize the linear relationship between the UVI and UVB irradiance at the crown of the head.

## Results

Measurements (the means of measurements taken at the eight azimuths) obtained from the sensor at the crown of the head and from those in the eye sockets at each hour were compared. Right and left eyes received equivalent intensities of UVB, so the values of the central socket sensor in the left eye were used as the ocular irradiance for calculation. Solar culmination on each measurement date occurred between 11:00 AM and 12:00 PM.

**Figure 5.**
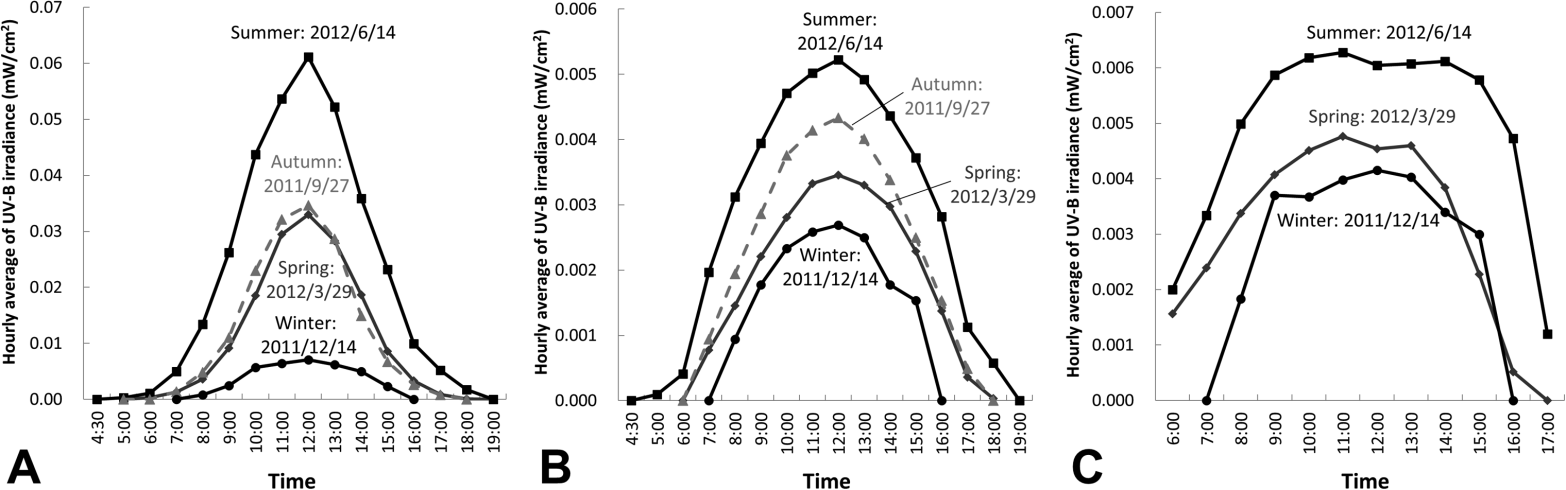
Hourly variation of UV exposure on each measurement day. (A) The eight-azimuth mean UV exposure at the crown. (B) The eight-azimuth mean UV exposure intensity at the eye. (C) The UV exposure at the eye when facing the sun.

Figure 5 shows the hourly variation of UV exposure on each measurement day. On the crown of the head, the mean UV irradiance at the eight azimuths increased with solar culmination (Fig. 5A). In summer, the UVB irradiance was 0.062 mW/cm^2^, corresponding to a UVI level of 7 at the time of culmination; in winter, it was 0.007 mW/cm^2^, corresponding to a UVI level of 1 at the time of culmination. The spring and autumn values were almost the same, at approximately 0.035 mW/cm^2^, corresponding to a UVI level of 5. Figure 5B shows the hourly variation of ocular UVB exposure (eight-azimuth mean) on each measurement date, ranging from approximately 9% to 38% of that to the crown at culmination time. Plotting the data revealed a bell-shaped curve with a peak at the time of maximum culmination. When the mannequin was facing the sun, the plotted data showed a bimodal curve for spring and summer, with twin peak values at 11 a.m. and from 1:00 PM to 2:00 PM, which differs from a previous report^29^ indicating that ocular UVB exposure when facing the sun was decreased at around noon (Fig. 5C). In winter, however, exposure varied little between 9:00 AM and 3:00 PM.

**Figure 6.**
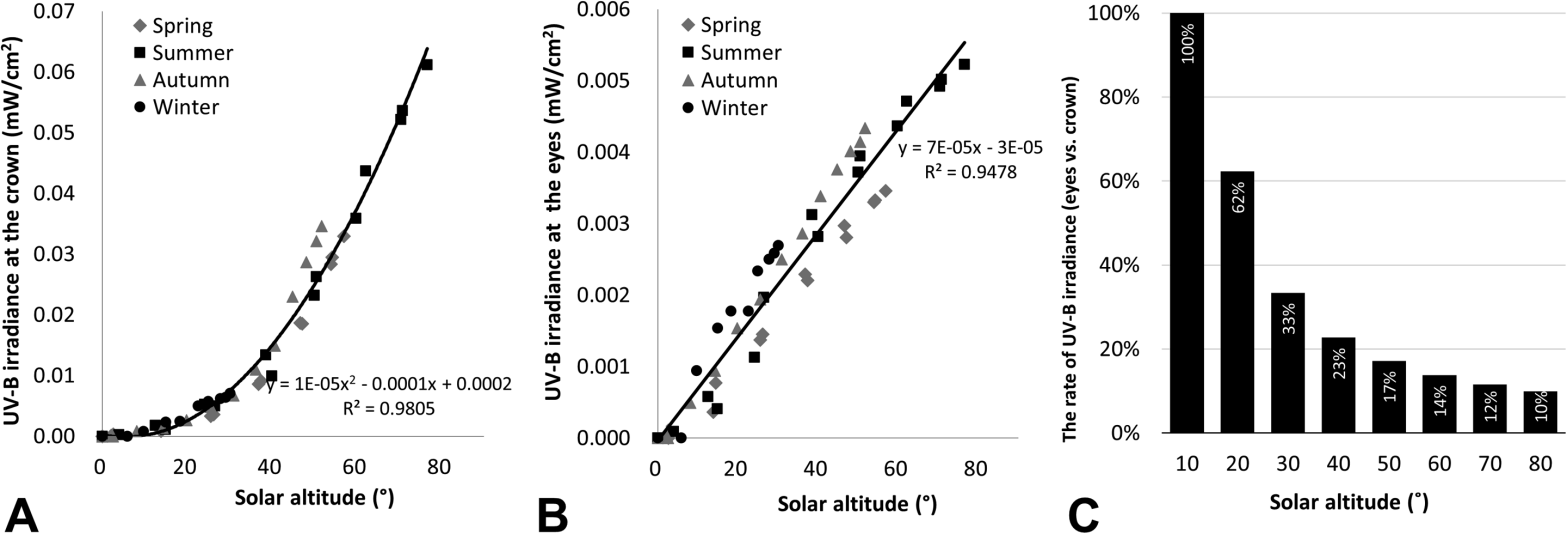
The eight-azimuth mean UVB irradiance by solar altitude: (A) at the crown, and (B) at the eye.

Figure 6 shows the relationship between crown and ocular UV exposure intensities (eight-azimuth mean) by solar altitude. Regardless of time and season, at the same solar altitude crown and ocular irradiances were comparable, and both increased at a rate similar to that for solar altitude. The correlation of solar altitude and ocular UV exposure was represented as a linear curve (Fig. 6B). Ocular exposure under low solar altitudes of 20° to 30° was 33% to 62% of crown exposure, but under solar altitudes above 50° it was approximately 10% to 17% (Figs. 6A, 6C).

Ocular UV exposures expressed as percentages of crown exposure intensities under maximum solar culmination by season were as follows: summer, 8.5%; spring and autumn, 10.5%; and winter, 38.2%. Table 2 shows these values by solar altitude in summer, revealing that as solar altitude decreased relative ocular UV exposure tended to increase.

**Table 2.** Ocular UV Exposure as Percentage of Crown Exposure by Solar Altitude in Summer

	Solar Altitude						
	70°	60°	50°	40°	30°	20°	10°
Relative ocular UV exposure	9.4%	12.2%	16.0%	28.4%	39.7%	37.8%	32.8%

**Figure 7.**
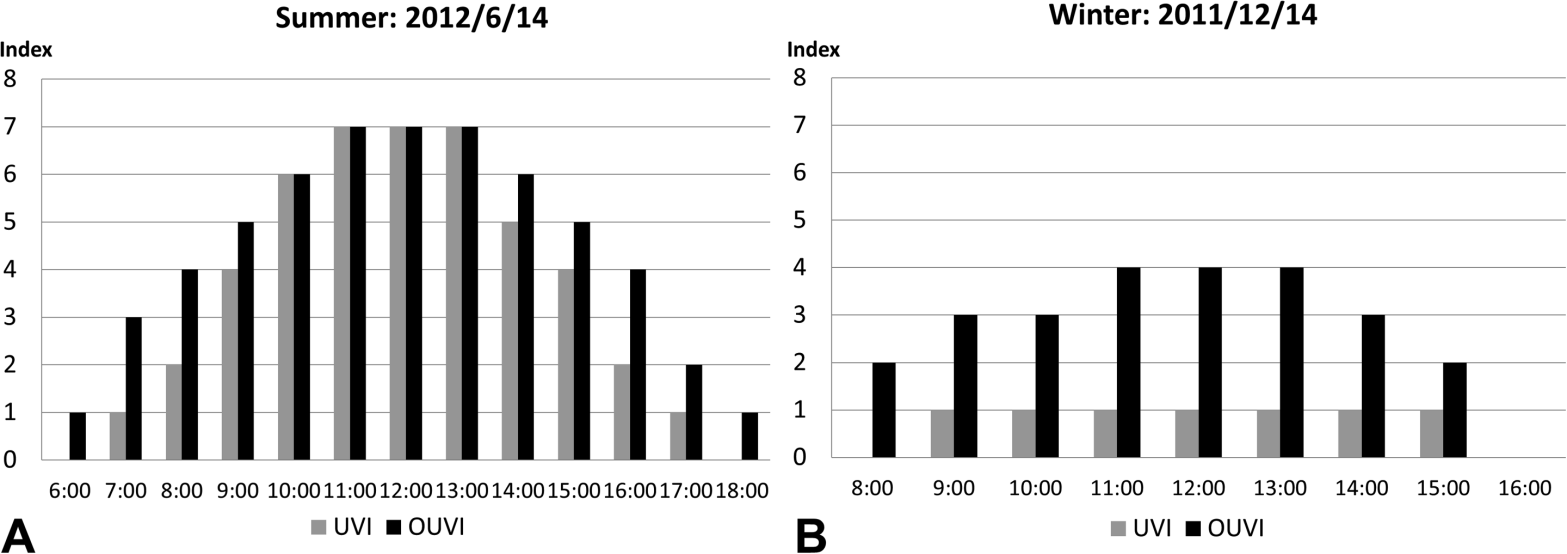
UVI and OUVI levels at the eight-azimuth mean: (A) summer (June 14, 2012), and (B) winter (December 14, 2011).

Figure 7 compares the UVI published by the Japan Meteorological Agency and the OUVI (eight-azimuth mean) determined from data obtained in summer (June 14, 2012) and winter (December 14, 2011). Due to the impact of scattering rays, OUVI levels approach UVI levels around midday in summer, when both solar altitude and UVI levels are high—for example, 76.7° (Fig. 3) and level 7 (Fig. 7A), respectively. Throughout the day in summer, solar altitudes and UVI levels remained low in the morning and evening, and the OUVI level was also low in the early morning and evening, but it was quite high (above level 4) from 8:00 AM to 4:00 PM. In contrast, in winter, when both solar altitude and UVI levels are low—such as 30.3° (Fig. 3) and level 1 at noon (Fig. 7A)—the OUVI level remained at around 4 (Fig. 7B), and OUVI levels were much higher than UVI levels throughout the day.

Table 3 shows the hourly OUVI levels determined from the eight-azimuth mean ocular UVB irradiances and, in parentheses, from UVB irradiances when facing the sun for each season. The OUVI level was at least 6 during the period from 10 AM to 2 PM on a clear-sky day in summer, which represents a relatively strong ocular UV irradiance. When the mannequin was facing the sun, the OUVI level remained above 6 from 8:00 AM to 4:00 PM; from 9:00 AM to 3:00 PM, the level was 8. In spring and autumn, the levels determined from the eight-azimuth mean ocular UVB intensities remained below 6, but when the mannequin was facing the sun the level was 6 from 11:00 AM to 2:00 PM. In winter, the maximum level determined from the eight-azimuth mean ocular UVB intensities was 4, and when the mannequin was facing the sun the level was 6 at 12:00 PM.

**Table 3.** Hourly OUVI Levels by Season^a^

	AM						PM						
	6:00	7:00	8:00	9:00	10:00	11:00	12:00	1:00	2:00	3:00	4:00	5:00	6:00
Summer	1 (1)	3 (5)	4 (7)	5 (8)	6 (8)	7 (8)	7 (8)	7 (8)	6 (8)	5 (8)	4 (6)	2 (2)	1 (1)
Spring and autumn	1 (1)	1 (2)	2 (3)	3 (5)	4 (5)	5 (6)	5 (6)	5 (6)	4 (6)	3 (5)	2 (3)	1 (1)	—
Winter	—	—	2 (3)	3 (5)	3 (5)	4 (5)	4 (6)	4 (5)	3 (5)	2 (4)	—	—	—

^a^Hourly OUVI levels determined from the eight-azimuth mean ocular UVB irradiances and, in parentheses, from UVB irradiances when facing the sun.

## Discussion

We have previously investigated ocular UV exposure,^29^^,^^30^ and in the present study we found that UVI levels measured in real time using the UVB sensor on the crown of the mannequin head were highly correlated with UVI. From the mean values of ocular UVB irradiance at the eight azimuths, we determined OUVI levels and compared them with UVI levels for daily and seasonal variation. We found large daily and seasonal variation in UVI levels but not in OUVI levels.

**Figure 8.**
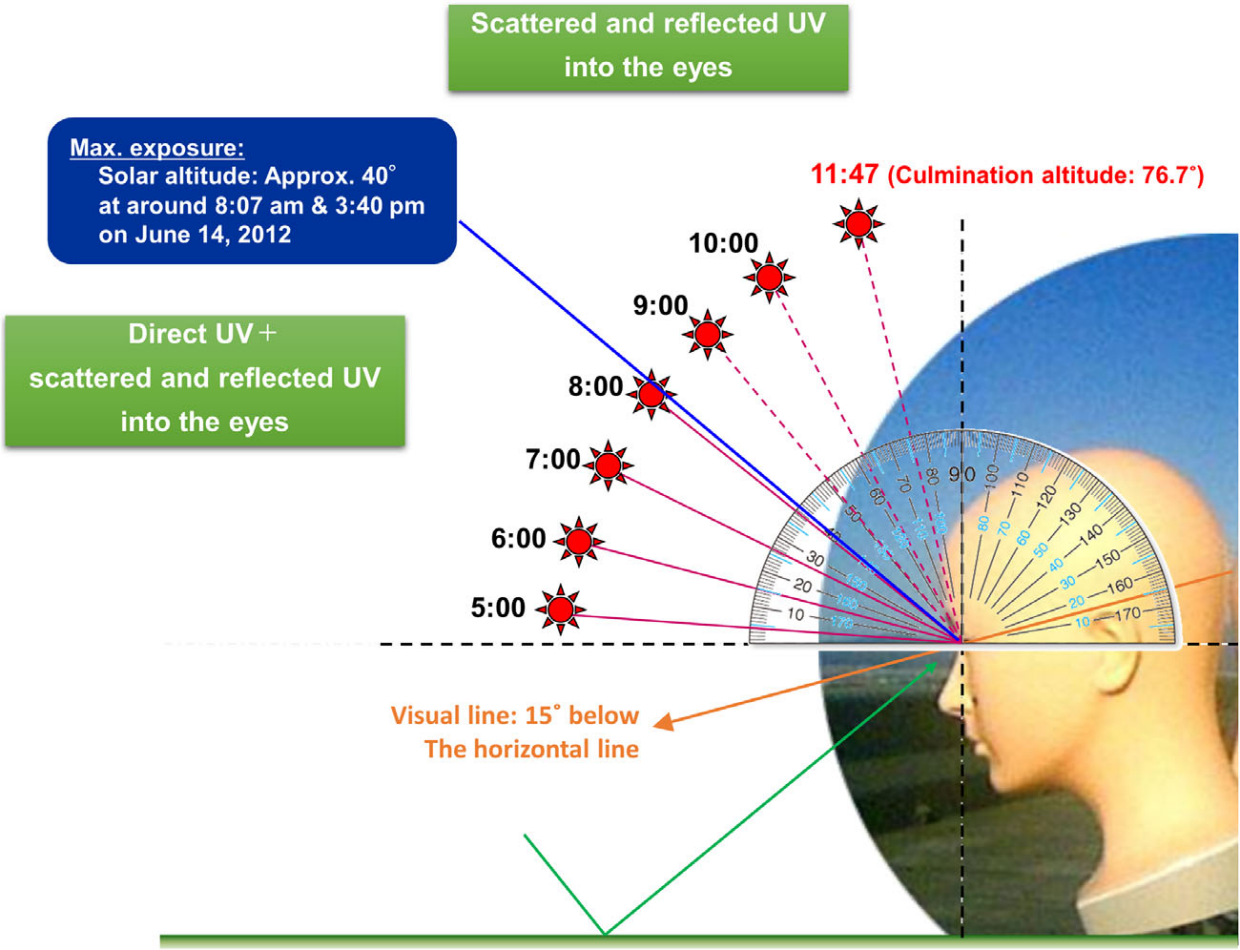
Solar altitude and UV exposure to the eye on June 14, 2012, in Kanazawa, Japan.

Compared with summer, exposure to the crown of the head was significantly lower in winter when the sun altitude was lower; however, exposure to the eye was slightly higher. This may be because the UV sensor on the crown mainly measured overhead solar UV and those on the eyes were influenced by the horizontal effects of the scattering component of UVB in winter when the sun altitude was lower. When the mannequin faced the sun (summer, spring, and autumn), ocular exposure slightly decreased around the time of maximum solar culmination but then increased again, so there were two peak times of high irradiance (Fig. 5C). This finding is consistent with those of previous reports,^29^^,^^31^ which have shown remarkably steeper bimodal peaks in mainly UVA (315–400 nm). In the present study, only UVB energy in the range of wavelength band from 280 to 310 nm was measured, so the slightly shallower observed bimodal peak irradiance on either side of maximum solar culmination might be due to the difference in the measured wavelength, which was shorter in our study. It could also have resulted from slightly different angular response (field of view) of the detector, and the influence of scattered light might have been stronger than that of direct light. In addition, ocular UV exposure, when the mannequin was facing the sun, was maximum when solar altitude was around 40°. This study used the face shape of an adult Japanese female; thus, when the solar altitude was high, direct rays from the sun did not reach the eyes of the mannequin because they were safeguarded by the forehead and eyelids. Mainly scattered light from the sky and reflected light from the ground and other surfaces reached the eyes (Fig. 8). This explains why ocular UV exposure when one is facing the sun would decrease during the period of maximum solar culmination; however, when the solar altitude is around 40°, ocular UV exposure is high, because direct rays from the sun are added to the scattered light from the sky and reflected light from the ground and other surfaces. In winter, under low culmination altitudes of around 30°, ocular exposure from direct sunlight when the mannequin faced the sun was strong throughout the day and reached maximum at solar culmination (Fig. 5C). It would be appropriate to predict the deleterious effects of UVA exposure; however, we included only UVB wavelengths in this study because UVB exposure is associated with severe damage to the human body, including DNA damage,^3^^,^^36^^–^^40^ and UVB is more scattered than UVA, affecting eyes even when one is not facing the sun. For this reason and because UVB exposure to the crown of the head showed a high correlation with UVI, we investigated OUVI using UVB wavelengths.

We took the mean irradiance values from the eight azimuths to represent ocular UV exposure in daily life, when people are mobile and often face in different directions. In contrast with facing the sun, when the sun is shining on us from a position at our side or behind us, the influence of direct sunlight to our eyes is small in proportion to that of scattered light. The eyes have a greater proportion of exposure to scattered UV than does the crown, which is under direct UV light.

Here we propose using the OUVI to measure the risk of ocular damage from UV exposure. We compared measurements of UVB at the eye with those at the crown of the head because we confirmed that those at the crown of the head correlated highly with UVI levels. As ocular exposure was approximately 1/10 that to the crown, the values of 10-fold ocular UVB exposure were used and defined as the OUVI with a linear scale from 1 to 13+. Because UVI levels correlate with the severity of skin erythema,^41^ in future studies we will assess the relationship between OUVI levels and severity of acute diseases such as conjunctivitis and keratitis. In summer, the UVI and OUVI levels were equivalent when the solar altitude was high, from 10 AM to 1 PM, as ocular UV exposure is strongly affected by sunlight scattering. But, when the solar altitude was low in the morning or evening, the UVI level was less than the OUVI level, due to direct rays into the eyes. In winter, the UVI level remained low at 1, because the solar altitude was low throughout the day, and the OUVI level remained above that of the UVI. These findings revealed a characteristic difference between the two indices, UVI and OUVI. Another characteristic difference concerns solar altitude. OUVI levels differ from UVI levels by solar altitude in every season. Even when a person is not facing directly into the sun, some direct rays enter the eyes from the side of the head, and some scattering and reflecting rays reach the eyes from the temporal side even when the person is wearing spectacles or sunglasses, which is a feature that may be involved in pterygium.^42^^–^^44^ Therefore, even when a person is not directly facing the sun, proper eye protection that includes peripheral protection from UV is necessary.

The OUVI was created using UV data obtained on fine or clear-sky days, but the OUVI level would be expected to change with weather and under different environments.^27^ A comparison of OUVI levels determined under a clear sky (June 14, 2012) and under a cloudy sky (June 11, 2012) is provided in Table 4. The measurement location and equipment were the same, and solar altitudes were presumed to be identical because measurement times differed by only 3 days. Under a clear sky, the OUVI level was high in the morning and the evening, but when the sky was cloudy it remained low. In particular, when people turn to face the sun, OUVI levels would be high due to direct sunlight; however, under a cloudy sky, when someone is facing in the direction of the sun the OUVI level would be similar to that determined by the mean of the eight azimuths. This indicates that direct solar rays are blocked by clouds, and their influence in the morning and evening decreases. However, we found that, around culmination time, the influence of direct sunlight was diminished even under a clear sky, and OUVI levels remained the same regardless of clear sky or cloudy weather. Accordingly, it would appear that weather and environmental differences can affect OUVI levels even at the same solar altitude.

**Table 4.** Survey Conditions and Hourly OUVI Levels in Summer^a^

Season	Date			Weather		Maximum Temperature (°C)		Sunlight (h)		Culmination Altitude (°)		Maximum UVI	
Summer	June 11, 2012			Cloudy		25.1		3.1		76.5		7	
Summer	June 14, 2012			Clear sky		26.1		13.4		76.7		7	

	AM						PM						
	6:00	7:00	8:00	9:00	10:00	11:00	12:00	1:00	2:00	3:00	4:00	5:00	6:00

June 14, 2012 (summer, clear sky)	1 (1)	3 (5)	4 (7)	5 (8)	6 (8)	7 (8)	7 (8)	7 (8)	6 (8)	5 (8)	4 (6)	2 (2)	1 (1)
June 11, 2012 (summer, cloudy sky)	1 (1)	2 (3)	3 (3)	3 (4)	4 (5)	6 (7)	7 (8)	6 (7)	6 (7)	2 (3)	2 (2)	1 (1)	1 (1)

^a^Hourly OUVI levels determined from the eight-azimuth mean ocular UVB irradiances and, in parentheses, from UVB irradiances when facing the sun.

This study had several limitations. The facial shape of the mannequin used in this study was characteristic of an adult Japanese female and not of other ethnic groups worldwide. Also, even among Asians, the mannequin facial shape differed slightly from that employed in the study by Hu et al.^31^ Thus, our results cannot be generalized to the entire human race, adults or children. In addition, the UV measurements were performed using a mannequin sited on a urethane-coated concrete roof, which has a UV reflectance of around 10%.^29^ Sliney^33^^,^^45^ found that masonry, concrete, and asphalt (e.g., roadways) generally reflect 8% to 10%, which was true for our 10% surface reflectance. He argued that much of UVB ocular exposure is due to ground reflection and not from overhead and that facial structure is less important than position of the upper lid. It has been also reported that terrain reflectance is more important than other factors, such as elevation from sea level to 8000 feet. In addition, solar UVB reflectance varies from around 1% for green mountain grassland to 88% for fresh snow. Although a urethane-coated roof reflects relatively little visible light, a reflectance of about 10% is representative of black asphalt and concrete pavement,^45^ which are fairly ubiquitous, thus representative of most commonly encountered outdoor environments. However, there is a limit to generalizing the results of this or any similar study to all environments. In the future, additional measurements in different environments (e.g., snow surfaces or white beaches with high UV reflectance, cloudy rather than clear skies) using mannequin models representing different ethnicities, both adults and children, should improve the generalizability of the OUVI as an indicator of ocular UV exposure.

## Conclusions

The OUVI differs from the UVI due to its inclusion of factors regarding the change of ocular UV exposure with solar altitude, the influence of dispersion and reflection, and the influence of season, weather, and environmental factors; for example, there may be a need to add a multiplicative factor of six- or sevenfold when snow is the ground surface. The UVI does not provide sufficient warning about the risks of ocular UV damage. The proposed OUVI is useful for warning the public about ocular UV exposure and can serve as a tool for research into UV-induced ocular diseases, such as cataract and pterygium.

## Figures and Tables

**Appendix A.**
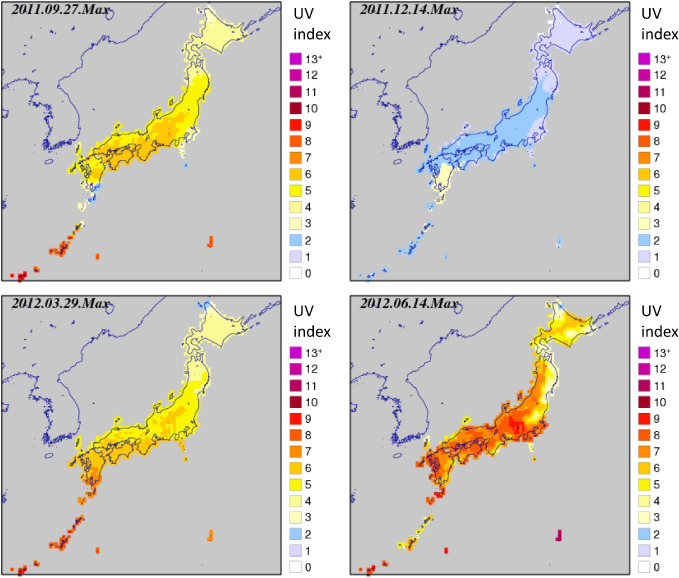
Maps of Japan indicating the maximum UV index data on dates of the study (Japan Meteorological Agency, http://www.data.jma.go.jp/gmd/env/uvhp/link_uvindex_ana.html).

**Appendix B.**
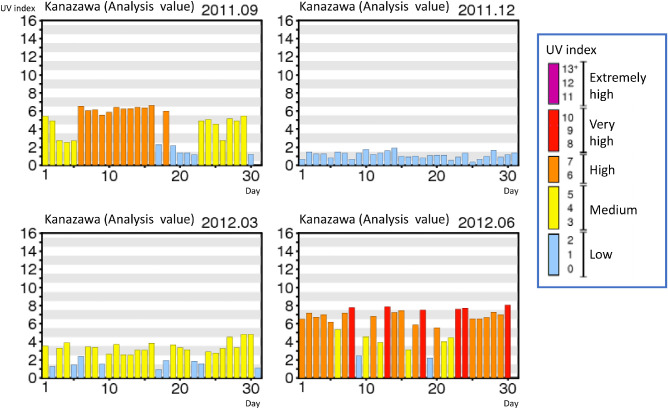
Charts showing maximum UV index data (analysis value) in Kanazawa for the months when the study was conducted (Japan Meteorological Agency, https://www.data.jma.go.jp/gmd/env/uvhp/link_uvindex_ana_chiten.html).

**Appendix C.**
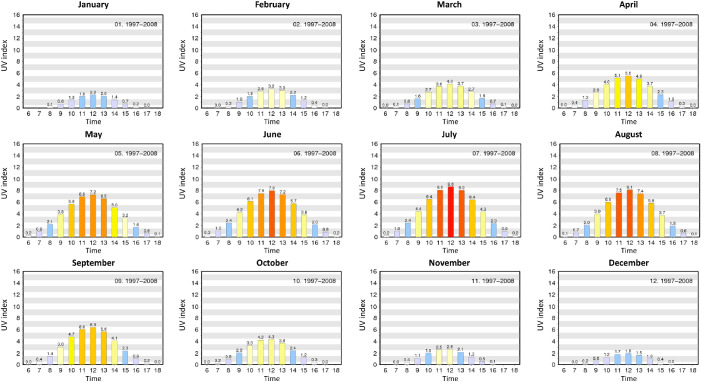
Charts showing monthly mean UV index values from 1997 to 2008 in fine weather (Japan Meteorological Agency, https://www.data.jma.go.jp/gmd/env/uvhp/link_uvindex_norm_hour.html).

**Appendix D.**
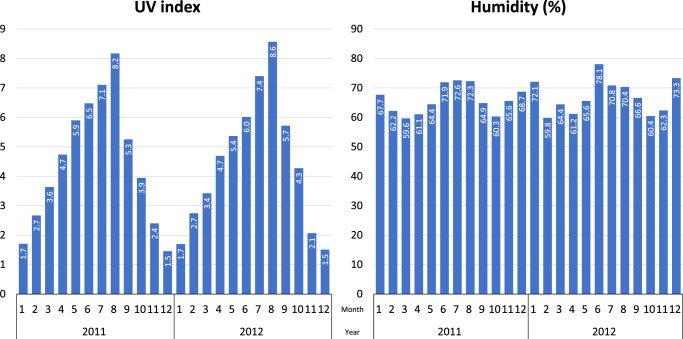
Bar graphs showing monthly noon mean values of the UV index and humidity at Kanazawa (2011–2012) using the NASA Giovanni system (https://giovanni.gsfc.nasa.gov/giovanni/).
